# Digitizing UK analogue magnetogram records from large geomagnetic storms of the past two centuries

**DOI:** 10.1002/gdj3.151

**Published:** 2022-03-28

**Authors:** Ciaran D. Beggan, Eliot Eaton, Eleanor Maume, Ellen Clarke, John Williamson, Thomas Humphries

**Affiliations:** ^1^ 41819 British Geological Survey Edinburgh UK

**Keywords:** analogue magnetogram, digitization, geomagnetism, observatories

## Abstract

Continuous geomagnetic records of the strength and direction of the Earth's field at the surface extend back to the 1840s. Over the past two centuries, eight observatories have existed in the United Kingdom, which measured the daily field variations using light‐sensitive photographic paper to produce analogue magnetograms. Around 350,000 magnetograms have been digitally photographed at high resolution. However, converting the traces to digital values is difficult and time consuming as the magnetograms can have over‐lapping lines, low quality recordings and obscure metadata for conversion to SI units. We discuss our approach to digitizing the traces from large geomagnetic storms and highlight some of the issues to be aware of when capturing magnetic information from analogue measurements. These include cross‐checking the final digitized values with the recorded hourly mean values from observatory year books and comparing several observatory records for the same storm to catch errors such as sign inversions or incorrect ‘wrap‐around’ of data on the paper records.

## Graphical abstract

Continuous geomagnetic records of the strength and direction of the Earth's field at the surface extend back to the 1840s of which 350,000 magnetograms have been digitally photographed at high‐resolution. Converting the traces to digital values is difficult and time consuming as the magnetograms can have over‐lapping lines, low quality recordings and obscure metadata for conversion to SI units. We show how to digitise the traces from large geomagnetic storms and highlight issues when capturing magnetic information from analogue measurements.

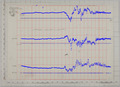

## 1 INTRODUCTION

In many areas of geophysical study, long time series of measurements exist in analogue form on photographic paper, in journals or as published tables. The conversion of analogue records to digital values is highly advantageous; for example, allowing modern computational techniques and analysis to be applied. Campaigns to digitize temperature or climate‐related measurements have been very successful especially with the recruitment of keen citizen scientists to help manually extract numbers from old or distressed paper records where optical character recognition technology struggles (Ryan et al., 2021; Skrynyk et al., 2021). As with climate records, geomagnetism has a very long history of observations available. Declination measurements from the 16th century exist in some locations (Alexandrescu et al., 1996; Barraclough et al., 2000; Malin et al., 1981) and from the era of exploration and sail, between the 15th and 20th centuries, high‐quality ship navigation records have been used to constrain the shape of the Earth's magnetic field back to 1590 (Jackson et al., 2000).

Prior to the 1830's there were no absolute measurements of the strength of the field though relative variations between sites could be deduced (Enebakk, 2014). In 1832, Carl Frederik Gauss invented a method of measuring total field intensity (Garland, 1979) and in this era of exploration, a great ‘magnetic crusade’ was embarked upon to understand the Earth's magnetic field by making measurements around the world (Cawood, 1979; Collier, 2014; Sabine, 1849). A legacy of this crusade was the establishment of many permanent geomagnetic observatories, some of which have lasted in one form or another for almost two centuries.

In Greenwich, London (UK) an initial dedicated manual observation programme was set up in 1838, with continuous recording instrumentation on photo‐sensitive paper beginning less than a decade later (Brooke & Airy, 1847). A rival observatory in Kew Gardens (around 20 km west) was later established in the 1850s. Further public and private observatories were set up across the United Kingdom, with Hartland being the most recently established in the International Geophysical Year of 1957. Overall, eight permanent ground observatories have existed since 1836 (Table 1) though there have been many more temporary ones (Kerridge, 2007). At present, three observatories are in operation in Britain (Eskdalemuir, Hartland and Lerwick) providing digital minute‐mean data since 1983 and 1‐second values since 2012 (Clarke et al., 2013). The UK observatory records held by the British Geological Survey (BGS) at present surpass 175 years of continuous recording.

**TABLE 1:** Available analogue magnetogram records by observatory

Observatory	Years available (inclusive)
Abinger, Surrey	1924–1957
Eskdalemuir, Dumfries and Galloway^a^	1908–1982
Falmouth, Cornwall	1887–1912
Greenwich, London	1836–1926
Hartland, Devon^a^	1957–1982
Kew, London	1857–1924
Lerwick, Shetland^a^	1922–1982
Stonyhurst, Lancashire	1866–1973

^a^ Digital from 1983 onward.

Although almost 40 years of data are held digitally, this leaves over 130 years of magnetic measurements confined to analogue paper records. If we were to unlock this resource it would allow magnetic field variation for around 11 solar cycles to be studied in greater detail than currently possible through coarser magnetic proxies like the *aa* magnetic activity index (e.g. Chapman et al., 2020) and allow better statistical analysis of extreme events (Rogers et al., 2020; Thomson et al., 2011). Though there are nascent tools being developed to undertake this task, it is not trivial to digitally extract the analogue traces from the magnetograms (e.g. Curto et al., 1996). In particular, large or extreme storms (which are of most interest for space weather hazard in the first instance) are often very difficult to decipher even by the trained eye. However, it is these events that are most attractive to extract first, rather than the far more common quiet‐time periods, as they provide insight to the potential effects of space weather on modern day technology (e.g. Hapgood, 2019).

In this paper, we describe our methodology for extracting analogue values of extreme geomagnetic storms from images of magnetograms and converting them to digital values of time and magnetic field strength in SI units. In Section 2, the database of magnetograms and the present state‐of‐the‐art digital capture methods are described. In Section 3, we discuss some of the limitations and drawbacks of the methodology and make an estimate of the uncertainty involved in the digitization process. In Section 4, we discuss the limitations associated with older records compared to modern measurements before concluding with our recommendations.

## 2 DATA DESCRIPTION AND DIGITIZATION

### 2.1 Magnetic data collections

Historic instrumentation at geomagnetic observatories was relatively simple in terms of the concept of operation. Depending on the component of the magnetic field, the measurement generally consisted of the observation of the behaviour of magnetized needles suspended by quartz fibres or clamped to the vertical. The instruments were kept in temperature‐controlled darkened chambers, often underground, where beams of collimated light reflected off mirrors on the needles to amplify small changes of position. To record continuous variations of the field, light‐sensitive paper was mounted on a rotating drum, which turned once per day capturing the trace of the reflected light. The traces were calibrated to standard (later SI) units of magnetic strength or angle depending on the instrument and component being measured, usually once per week using manual measurements to fix the absolute values. These established the baseline to which the variations were referenced. The paper on the drum was manually changed once per day and the magnetogram traces were photographically fixed before being analysed for hourly values or reduced to geomagnetic index values such as the three‐hourly K index for the observatory. These were later published in the official observatory yearbooks, often alongside meteorological and other geophysical observations. For further detail, Clilverd et al. (2018), Curto (2019) and Nevanlinna (1997) all offer explanations of the types of historic instrumentation used between 1890 and 1990 across the world.

The UK magnetic records consist of the magnetic variation of three components of the field; usually the horizontal and vertical force and the Declination angle (the deviation of the compass needle away from true North). The other components such as inclination angle can be computed from these measurements. The earliest continuous paper records began in the 1846 in Greenwich observatory in London. The dynamic range of the instruments was usually limited in order to capture small diurnal variations. During times of high magnetic activity, the needle would move out of its nominal limit and so a series of prisms were used to extend the range of the three elements recorded. The prisms were aligned so that as the light spot moved off the edge, another spot appeared at the other side, allowing amplitude to continue to be recorded (Newitt, 2007). However, this ‘wrap‐around’ of traces often makes it difficult to decipher the true variation of the field during large storms and during very large storms the trace is lost entirely as the light beam moved beyond the range of the paper drum. Over the centuries, the general techniques and instrumentation evolved relatively little until the widespread introduction of digital proton precession magnetometers and fluxgate magnetometers in the 1970s (Newitt, 2007; Primdahl, 1979). Due to their excellent accuracy and low maintenance costs they remain the primary sensor for continuous recording of the field at modern observatories (Jankowski & Sucksdorff, 1996).

**FIGURE 1:**
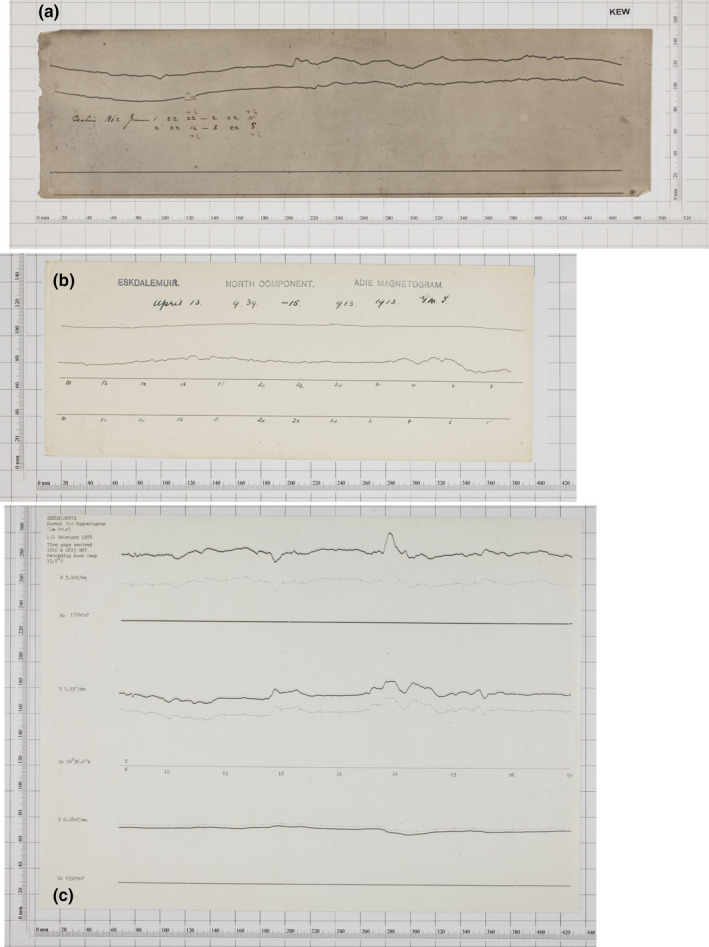
Example magnetograms: (a) Kew (Declination angle): 01 June 1861; (b) Eskdalemuir (Declination angle): 13 April 1912; (c) Eskdalemuir (Horizontal (H) Force, Declination angle and Vertical Force (Z)): 01 February 1978. Note only the lower panel provides SI metadata on the plot

In response to the threat of loss from degradation due to age and a desire to preserve and exploit old data, over the past decade a sustained effort has been made by the BGS to digitally photograph, archive and preserve the analogue paper records of magnetic field variation in the United Kingdom. Between 2007 and 2013, digital images of every available magnetogram were taken. To do this a fixed‐position high‐resolution digital camera (Canon D5 Mark2, 21 Megapixels with 60 mm macro lens) was used. Figure 1 shows an example of magnetogram images from three different dates. Panel (a) shows the Declination recorded at Kew on 01‐June 1862, (b) is the Declination at Eskdalemuir on 13/14 April 1912 and (c) illustrates the three components on 01 February 1978. The scale bars surrounding the magnetograms are in millimetres. In later years, after 1960, more information (in SI units) was placed on the magnetograms but in most cases, the scaling information is only found in the yearbooks of the particular observatory making completely automated extraction of the data awkward as the required metadata can lie in different documents. Whilst photographing the magnetograms, the entire observatory yearbook collection was also digitized using a Bookeye 3 Scanner at 300 dpi resolution. The timing information can also be difficult to extract precisely, as it is sometimes written on by hand rather than being printed (as in later years). It is also clear that different sizes of photographic paper were used over time.

Every magnetogram (front and back) dating from the mid‐19th century through to the digital era of geomagnetic recording is now available to search and view from the BGS website. A web service was also developed to enable access to the images as zoomable JPEG2000 format and is freely available to download in a format selected by the user. The digital archive contains around 350,000 magnetograms from the eight UK observatories.^1^ The scanned magnetograms are accompanied by the yearbooks from each observatory. These provide vital metadata and information on the observatory operations, observing equipment and observation methods required to interpret the magnetograms and other data. The yearbooks also provide other data, such as hourly mean values, which can be a useful cross‐check of the magnitude of the digitized magnetogram values.^2^

Throughout the lifespan of observatories in the United Kingdom, different coordinate systems have been used in the magnetograms to represent the vector of ground magnetic field. Historically, H, D and Z, are typically used to give the magnitude of Horizontal field strength, Declination and the vertical component respectively. Conversion between definitions of the vector ground magnetic field is given by X=Hcos(D), Y=Hsin(D), $I=\tan^{‐1}\left(Z/H\right)$ and $F=\sqrt{H^{2}+Z^{2}}$, where X and Y are geographic north and east, I is angle downwards from horizontal and F is total intensity.

### 2.2 Converting to digital values

Having captured the images of the magnetograms, the next step is to convert the analogue information and line traces on the scanned magnetograms to digital values. Unfortunately, there is, as yet, no simple or generally applicable technique or methodology available due to the unique configuration of the magnetograms from each observatory and the constant change of instrumentation calibration, baselines and recording processes over the years. Digitization thus requires suitable technical knowledge and experience in understanding the quirks of each observatory and the magnetogram recording system. For the present, the software limitations force us to focus our efforts on large or extreme geomagnetic storms, as these are of most interest in the context of space weather hazard (Hapgood, 2019; Tsurutani et al., 2003). Due to the varying form of historic magnetograms between years and observatories, we can only provide the general framework to digitize each magnetogram. Adjustments are usually required to fit the specific outputs from each observatory and for each set of recording instrumentation.

After searching for and trialling suitable software packages (including developing a bespoke image analyser), we chose Engauge Digitizer to convert the lines on the magnetogram images to digital values. Engauge Digitizer is a free open‐source programme that allows a user to import image files containing graphs, manually trace over a graph and output a text file containing the calibrated digitized coordinates of the graph. To use it, the image files must first be converted into a standard format (PNG, JPEG or TIFF) from the scanned JPEG2000 file format. Our method involves two main steps: capture the baseline and the variation of the field, and then correctly scale the digital units to magnetic field values and time using metadata from the magnetogram or yearbook values.

**FIGURE 2:**
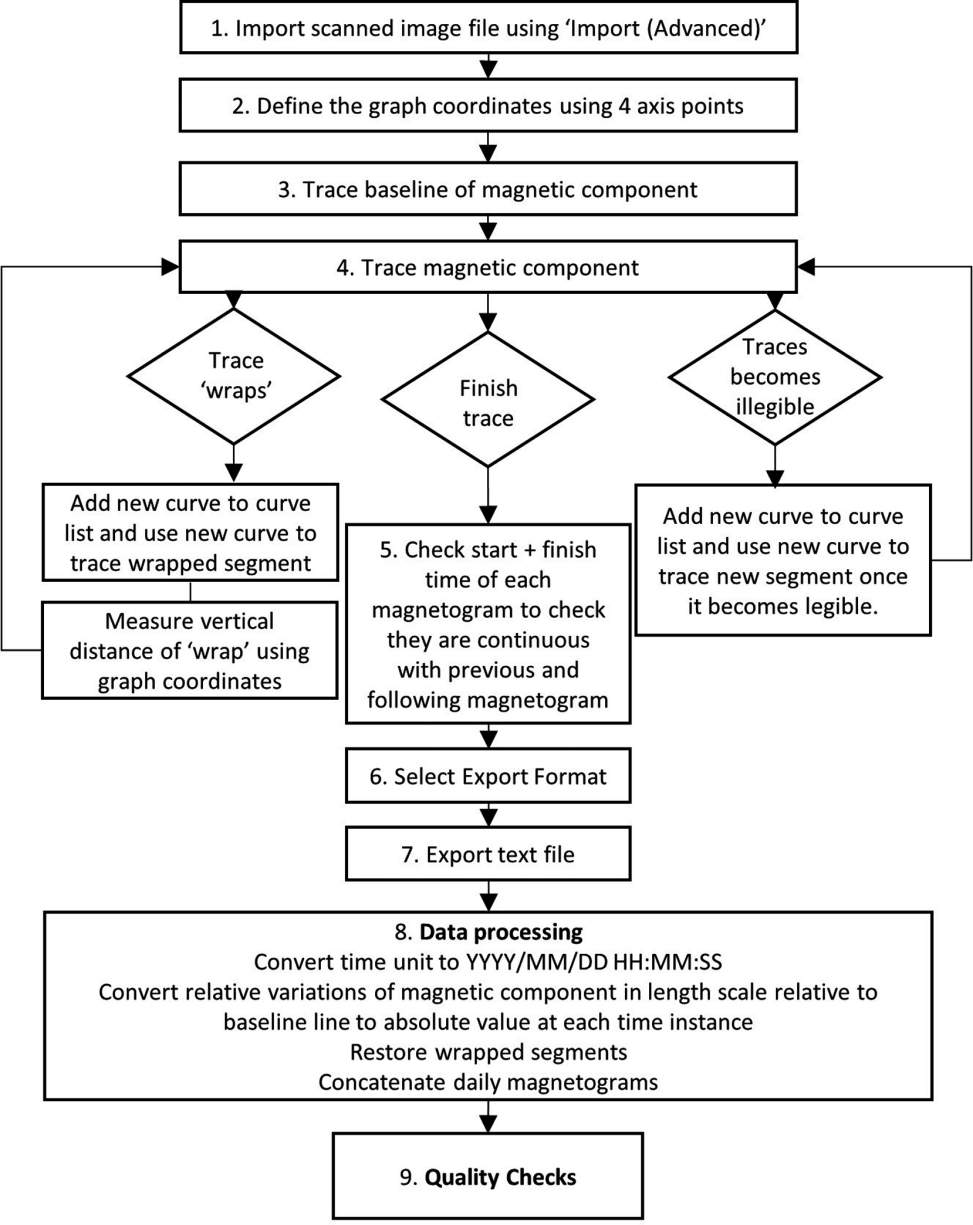
Flow chart describing process of digitizing analogue magnetograms

Figure 2 provides a flowchart of the steps involved in digitization. In detail, the image file is imported into the software using the ‘Import Advanced’ option in Engauge Digitizer. If the time axis of the magnetogram is not perpendicular to the length scale on the vertical axis, image pre‐processing is used to make the pair of coordinate axes perpendicular. After importing the image file, the graph coordinates must be defined to calibrate the coordinate system of the magnetogram relative to the image. Using the ‘Advanced’ import option allows the graph coordinates to be defined using 4 axis points, 2 on each axis. This option is selected as only one set of coordinates are defined on each axis. The vertical axis is defined using the length scale of the scale bar in millimetres included in the image of the magnetogram. The vertical axis is defined using two points extending the range of the signal recorded. The horizontal time axis is defined using the hour marks for each component of the magnetogram. Note, if more than one component is included on the magnetogram, that in some cases the hour marks in each of the three components (H, D and Z) do not align, so need to be handled separately with an independent x‐axis. If the exported time series have gaps at the beginning and end of each day or extend longer than the time range of the magnetogram, this is an indication of an inaccurate definition of the time axis. To define the x‐axis with 2 points, use the earliest defined hour mark and the last defined hour mark as $x_{1}$ and $x_{2}$ respectively. For example, if defining the magnetogram using 09:00 am on 01 January 1945 and 09:00 am on 02 January 1945, $x_{1}$ = 9 and $x_{2}$ = 33. This eases computation post‐digitizing.

Defining the vertical scale in units of millimetres and the horizontal scale bar in units of time calibrates the output text file of the software. For most magnetograms there is a baseline plotted for each component for which the absolute value of the field is given (either on the photograph or in a year book). This is usually constant or with a small amount of linear change over a day. The time‐varying part of the field is scaled relative to the baseline value. A separate scale value for each component can often be found to allow conversion between millimetres and the SI unit (e.g. arc‐minutes or nT). To correctly scale each component, the amplitude of the time series is calculated using the relative distance between it and the baseline. Both the time series and baseline must be traced to give the absolute value of the component.

For each component, a scale factor is required to scale the digitized time series from units of millimetres to nT for Horizontal force and Vertical force, and arc‐minutes for Declination. Often, as shown in Figure 1c, the scale factor can be found on the scanned magnetogram itself. Alternatively, scale factors may be found in the historic yearbook for each observatory.

For each time instance, the relative difference between traces (in unit of mm) of baseline and magnetic component for each time instance is calculated. This is then multiplied by the scale factor to give magnetic component in SI units. The baseline value is then added to the relative difference at each time instance to arrive at the absolute value. An example is given here for the Horizontal field strength H at time instance i,

(1) $$H_{i}=\left(H1_{i}‐H0_{i}\right)\times C+B_{H}$$

where H1 is the traced horizontal component with units of mm relative to its vertical position, H0 is the traced baseline with units of mm relative to the vertical position, C is the scale factor to convert between length scale and magnetic units, and $B_{H}$ is the known baseline constant (in nT).

Often during a geomagnetic storm, rapid change in amplitude causes the quality of the trace to be illegible. If there is a gap where the data become illegible, we start a new curve when digitizing the remainder of the signal. This ensures that there are no values within the missing time period and the gap is not interpolated across. In later years, some observatories used a second (La Cour) magnetometer with a larger unit/mm scale allowing for a better approximation of the trace amplitude but with a loss of the higher frequency signals (e.g. Figure 6). Furthermore, during periods of heightened activity of a geomagnetic storm, the magnetogram ‘wrap‐around’ appears at the top or bottom of the paper or other side of trace. This part of the signal requires the addition of a constant to restore its amplitude. The magnitude of this constant is found (in length scale) by measuring the distance between neighbouring time instances in the trace where the wrap‐around occurs and then adding/subtracting this distance during processing of the text file to the separate curve used to digitize this section of the magnetic component. Again, if the larger scale magnetograms are available, they can be used to confirm the shape and amplitude of the trace. This is where significant errors in digitizing can occur if unable to correctly track the wrap‐around of the magnetogram.

The user can choose whether to fit a function to the user input points using linear or cubic spline interpolation. Engauge Digitizer can fit a curve to the user input points using a cubic spline interpolation though issues can occur if the manually selected points are too tightly spaced as spurious signals can be introduced by the curve‐fitting feature. If the signal is not visible for a section of the time series, a new variable must be started to avoid curve‐fitting across gaps in the signal. When exporting from Engauge Digitizer the ‘Export format’ option is used to determine the rate at which the interpolation between points is sampled. Choosing to interpolate the y‐axis at evenly spaced x‐axis values allows the user to determine the sample rate in units of decimal hours. The ‘Extrapolate outside endpoints’ option is not selected to ensure values are extracted only for periods in which magnetic data are present and non‐existent values are not created in periods where the trace is missing or illegible.

### 2.3 A modern digital storm

An obvious test of the fidelity of our digitization technique is to photograph and then digitize a modern storm, comparing the output to the measured values. The Halloween storm of 29‐31 October 2003 is one of the largest storms in the digital record and it caused damage to high voltage transformers in South Africa and Sweden for example (Pulkkinen et al., 2005).

The full field magnetograms for the 2003 storm with SI units and scale annotated on the page were printed onto A3 paper, then photographed in a similar manner to the analogue magnetograms and then digitized using Engauge Digitizer. The digitized values were sampled at three different average cadences. One minute is equivalent to the measured cadence whilst the 5 and 10 min averages were computed in order to examine the effect of digitizing at different time resolutions. The process is not particularly quick; we find that it takes between two hours to manually digitize a simple storm and up to eight hours for a very complex set of variations. However, there are no wrap‐arounds of the magnetogram in digital records. For older magnetograms though considerable care and attention is required when picking the peaks. Having tested with two different people we find that there can be slight variations due to marginal differences in the coordinate system setup and manual picking of points.

**FIGURE 3:**
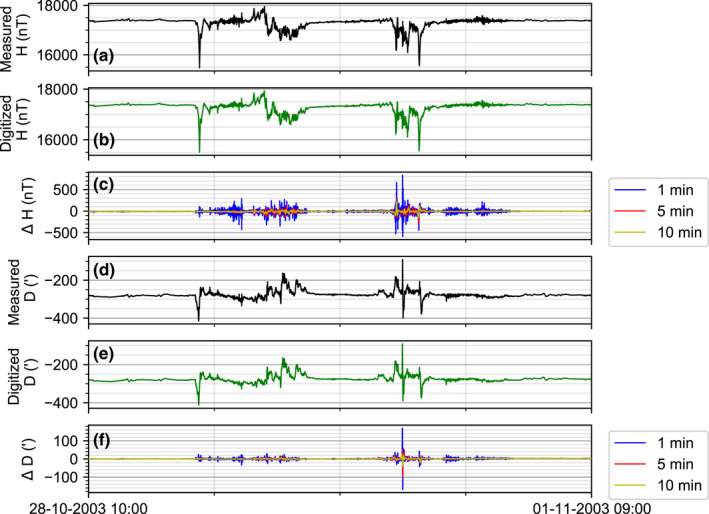
Comparison between digitized and recorded time series of October 2003 storm at Lerwick Observatory. (a) Measured horizontal field (b) digitized horizontal field strength. (c) Difference in horizontal field strength magnitude between recorded and digitized time series when sampled at 1 min intervals and then re‐sampled at 5 and 10 min intervals. (d) Measured Declination angle (in arc‐minutes) (e) digitized declination angle. (f) Difference in declination magnitude between recorded and digitized time series when sampled at 1 min intervals and then re‐sampled at 5 and 10 min intervals

Figure 3 shows the measured and digitized Horizontal (H) component and Declination angle (D) at Lerwick Observatory. During the storm there were two main phases of rapid variation: around midnight on the 29th October and noon of the 30th October. During the first phase, measured H falls to around 16,000 nT from 17,250 nT (panel a). The digitized version are similar. The differences between the digitized and measured H are relatively small (panel c), particularly where the magnetic variations are slow. The 1‐minute differences during this phase are around 10% (~100 nT) of the field values at most.

However, when the changes become more rapid a few hours later, the 1‐minute differences start to become larger. In the second phase of the storm on the 30th October, the fall in H is more rapid and the differences reach a similar size to the variation (|500| nT), which suggests the points picked are out of phase or a slight offset in time from the measured value has been introduced in the post‐processing. The errors are smaller with the 5 or 10 min average values as these smooth out the rapid variations. A similar pattern is seen in the Declination values (panels d, e, f). The comparison of the performance with a modern storm illustrates the likely uncertainties attached to manual digitization. The primary issue is correctly capturing the timing during high rates of change when the cadence is also high (e.g. 1 min). It also shows that even when the quality of the printed magnetogram is excellent and there are clear metadata available for scaling to time and SI units, it is difficult to correctly capture the data in periods of rapid variation.

## 3 HISTORIC GEOMAGNETIC STORMS

### 3.1 March 1946

**FIGURE 4:**
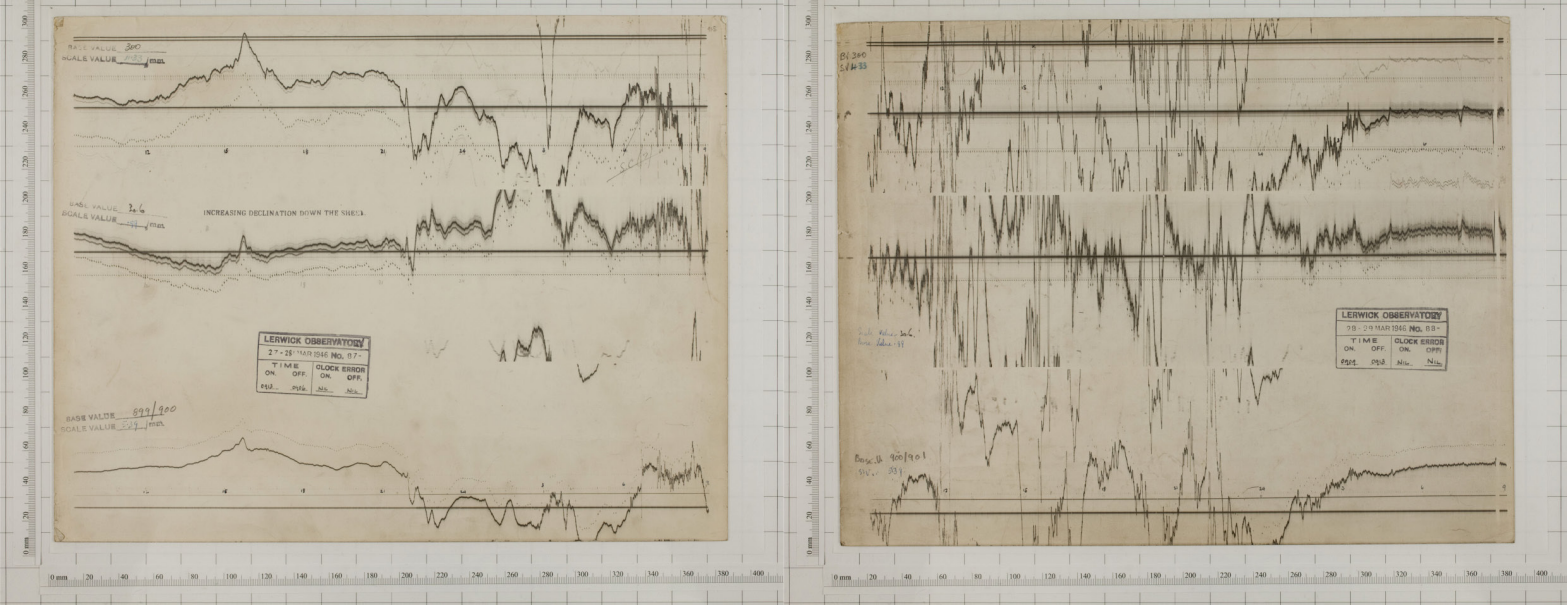
Magnetograms for Lerwick Observatory on the 27‐29th March 1946 for the H, D and Z components

The storm of the 27–29th March 1946 ranks as the seventh largest using the *aa* index (Hayakawa et al., 2020) and had an estimated minimum Dst index value of around −512 nT. It had unusually large horizontal (H) variations even at low latitudes and extensive aurora were visible for several days (Scott, 1946). Figure 4 shows the magnetograms recorded over the two day period from around 09:13 UT on 27th March to 09:13 UT on 29th March. The first part of the day is relatively quiet before the storm commences before 03:00 UT. The H component wraps around as does D, whilst Z wraps a few hours later. The wraps are confined to within each third of the magnetogram covered by the particular component. By 09:00 on the 28th the storm was in full swing and there are very large variations with dozens of wrap‐arounds in each component making extracting the values quite difficult. At 03:00 on the 29th the storm subsides and the variations are once again easy to follow, though now there are magnetospheric pulsations, which occur as small‐scale periodic features.

**FIGURE 5:**
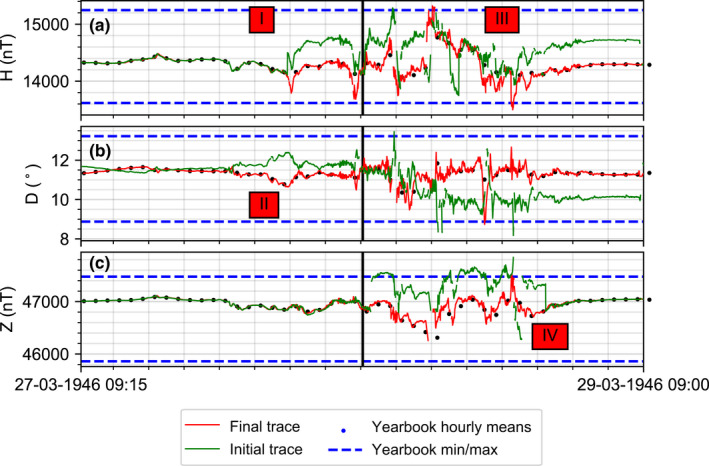
Lerwick March 1946: Example of common errors made when digitizing magnetograms. The green time series shows common mistakes made when digitizing photographic magnetograms, for example: (I) Incorrectly captured wrap‐around of the trace causing an offset. (II) Inverted application of scale factor. (III) Incorrect wrapping of a particular segment. (IV) Incorrect baseline partially applied in post‐processing. The red trace shows the corrected time series using the hourly mean and min/max values stated in the yearbook as a cross‐check. Vertical lines indicates the changeover of the paper between days

This magnetogram was digitized with care but whilst doing so a number of mistakes were made in the subsequent reconstruction. Figure 5 shows several examples of common errors made when extracting data from the magnetograms. In the figure, the red lines show the final extraction and post‐processing of the three components (H, D and Z) with the correct time series generated using both the magnetogram traces and the hourly mean values from the 1946 yearbook as a cross‐check. As expected the mean values fall within the red traces. The minimum and maximum of the storm (also noted in the yearbook) are further confirmation that the traces are within the expected ranges.

The green time series shows the initial trace constructed, which looked quite plausible. However, a number of mistakes had been made due to unclear behaviour of the recorded traces, ambiguous metadata and incorrect post‐processing. These were identified in the following manner:

- In the H component (panel A), the red box with label (I) shows where the first wrap‐around of the trace has introduced an offset, which was propagated through the remainder of the time series. The best check is to confirm that the magnetic field returns to its quiet level after the storm ends by extending the series to include quiet times before and after the storm
- In panel A, the box with label (III) indicates locations where the wrap‐around of the trace has not been correctly placed compared to the previous wrap‐around segment. Cross‐checking the trace with the hourly mean values can identify these errors.
- In the D component (panel B), the red box labelled (II) shows where the sign of the variation from the baseline is inverted and the values are wrongly recorded in the opposite sense in the post‐processing. Again, cross‐checking with the hourly mean values and other observatory records can identify these mistakes
- Panel C (Z component) shows an incorrect baseline offset added between two consecutive days as the digitization of each image was created separately. A comparison with the quiet‐time periods before and after the storm will identify this step if it is not visually obvious.

The figure also shows the minimum and maximum recorded values at Lerwick for the storm as described in the yearbook. This allows the extracted storm values to be compared to the manually estimated limits identified by the contemporary observers. The recommendation is made to cross‐check the final traces with other sources of information in yearbooks or traces from observatories reasonably close by in order to catch errors.

### 3.2 August 1972

The 4th/5th August 1972 storm occurs towards the end of the analogue era and so offers the opportunity to investigate how easily we can digitize a large storm with well‐preserved paper and clear metadata. This particular geomagnetic storm was associated with a very fast Coronal Mass Ejection (CME) from the Sun and a sharp initial impulse as the CME front interacted with the Earth's magnetic field. It was accompanied by a relatively small Dst index value (−125 nT) but very large rates of change of the magnetic field across the world including at mid‐ to low latitudes. One of the more unusual consequences of this storm was the near‐instantaneous unintended detonation of US Navy sea mines in Vietnam during a naval blockade (Knipp et al., 2018).

**FIGURE 6:**
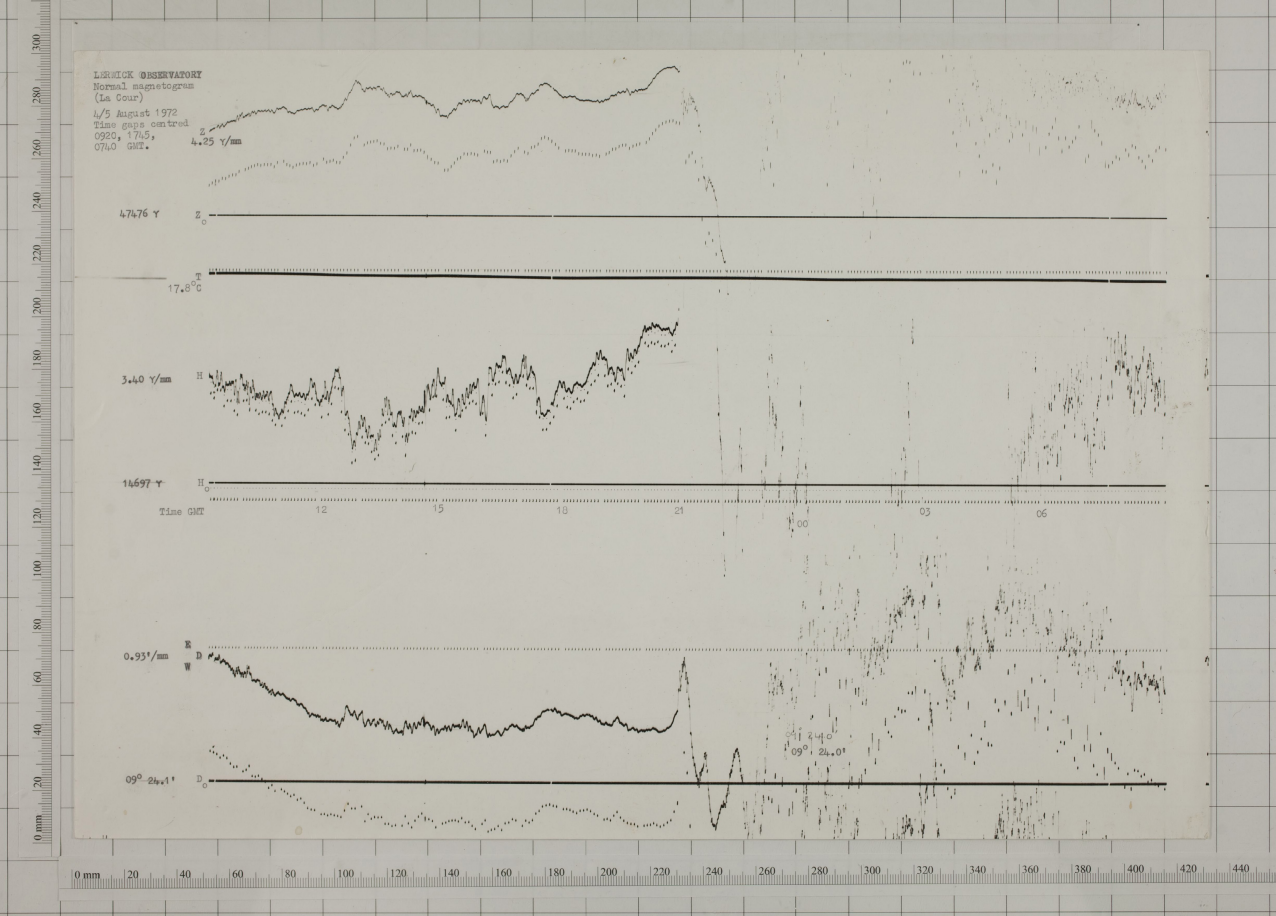
Image of the analogue magnetogram from the August 1972 storm recorded using the “La Cour” magnetometer at Lerwick Observatory on the 04/08/1972

Figure 6 shows the magnetogram^3^ from Lerwick observatory for the 4th August 1972 recorded on the ‘normal’ magnetogram system. The SI units and baseline scale (e.g. 4.25 nT/mm for Z, Z_0_ = 47,476 nT) are written onto the page making that part of the process simple. During the first part of the day the traces are clear and readily visible. At around 22:00 UT the storm commences, at which point the traces become very faint and difficult to read as they overlap and wrap‐around.

**FIGURE 7:**
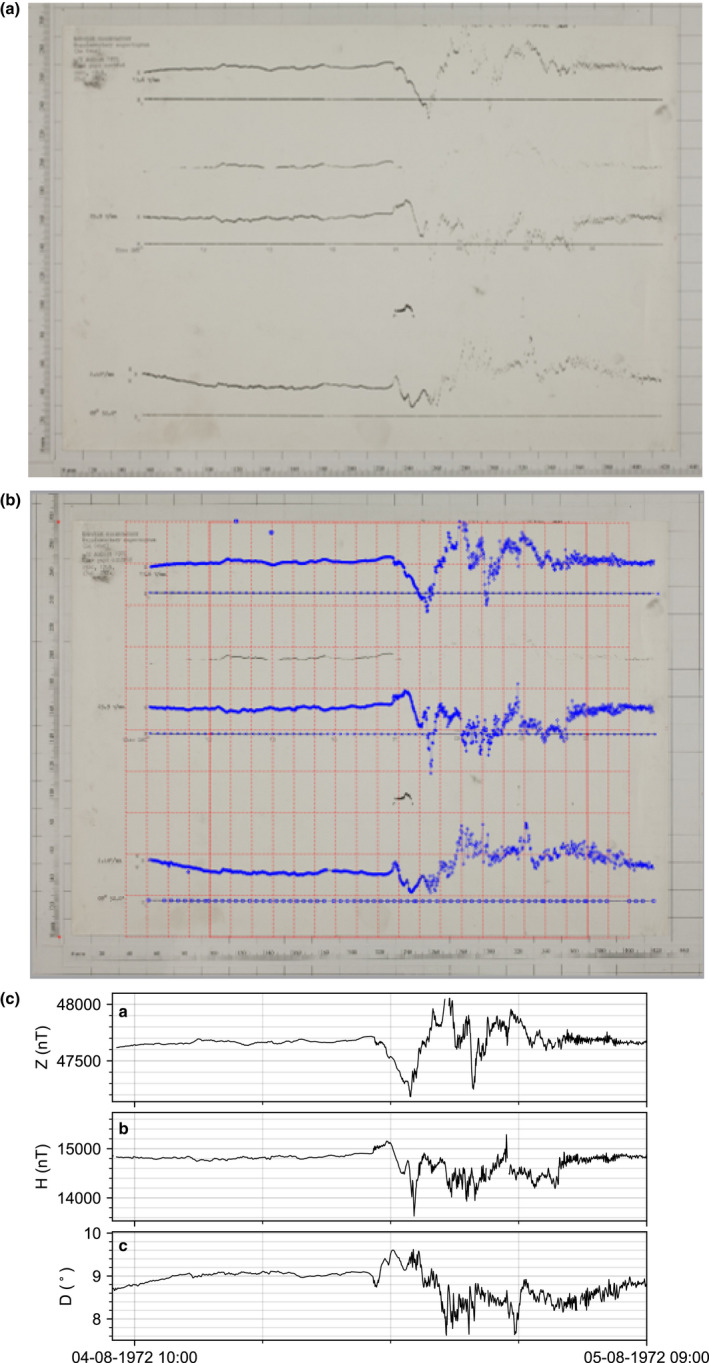
Digitizing the August 1972 storm. (a) Image of the supplementary analogue magnetogram from the August 1972 storm recorded at Lerwick Observatory on the 04/08/1972. (b) Screenshot of digitizing the supplementary analogue magnetogram from the August 1972 storm recorded at Lerwick Observatory on the 04/08/1972. (c) Digitized time series of the supplementary analogue magnetogram from the August 1972 storm recorded at Lerwick Observatory on the 04/08/1972

Fortunately, a second ‘supplementary’ recorder was running simultaneously with a lower sensitivity of 13.6 nT/mm. This allowed a better record of most intense part of the storm to be captured. Figure 7a shows the traces from the ‘supplementary’ recorder. The magnetogram^4^ captures the first part of the day clearly though again around 22:00 UT the traces become faint and hard to distinguish. The image was imported into Engauge Digitizer and the baselines and variations were manually traced (Figure 7b). These were converted into the correct time and SI units for analysis in 7c. Note that even with reasonably clean and clear magnetograms there are gaps in the H component of the storm in panel c.

**FIGURE 8:**
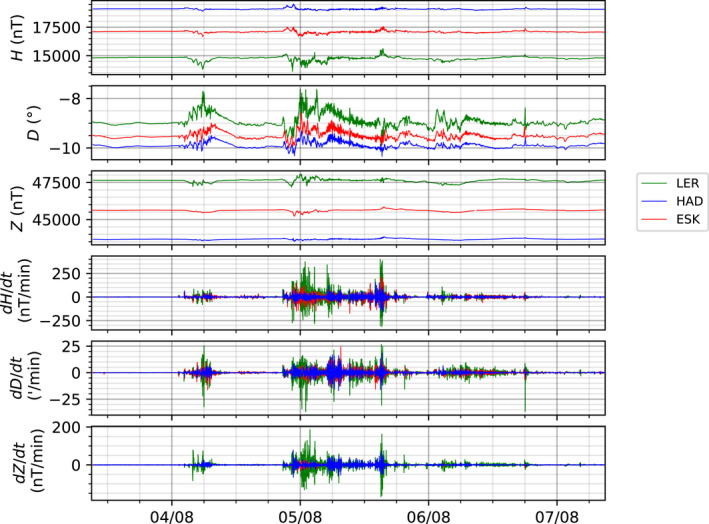
Final digital full field values from the three UK observatories for the August 1972 geomagnetic storm. Cadence is 1 min

The Eskdalemuir and Hartland observatory magnetograms were digitized in a similar manner. Figure 8 shows the benefit of digitizing the storm. The upper three panels plot the full field variations at three observatories. Once converted the data can now be readily manipulated, for example by computing the rate of change of the field at each minute. The dB/dt is often used as a proxy for the intensity of the storm and for a guide to the hazard posed from geomagnetically induced currents (Thomson et al., 2011). During the 1972 storm, the rate of change in Lerwick reached over 300 nT/min during the 5th of August according to the analogue records.

## 4 DISCUSSION

The 1946 magnetogram illustrated common issues when working with analogue records. Some of the problems relate to the instrumentation that was used at the time being unable to capture large amplitudes whilst others are from ambiguities arising from the post‐processing that must be identified from experience.

One of the more pertinent issues is to observe that old magnetograms and instrumentation do not faithfully record fast variations or extremes of magnetic field intensity particularly well. As can be seen, during periods of rapid field change, the traces become lighter and more diffuse as the focused light beam does not persist or dwell long enough on the photographic paper. Drum rotation speed was typically set at around 1 inch or 25 mm per hour so rapid changes would not resolve well in time either if the variations are on the sub‐minute scale.

In addition, the response of the instrumentation (i.e. suspended needles) would have been damped by the tension of the thread (usually drawn quartz) or the response time of the reaction of the field itself, which would have depended on the mass of the needle. Very rapid changes and large deviations are, therefore, not well recorded as the needle's motion would move out of phase with the true variation if it was shorter than the oscillation period (usually a few seconds). This impacts on our ability to compare analogue records to modern ones as digital instruments are very agile and respond in microseconds to variations of the field. Ideally, we would recreate a set of older instruments to infer a transfer function between the variation of the field and the response of the instrument.

However, for many large storms recorded in the UK, we can benefit from multiple observations at a number of different observatories. This is an important advantage as we can compare observatories with ambiguous traces with many wrap‐arounds to other available observatories at lower latitudes. Higher latitude observatories (e.g. Lerwick) are more likely to experience off‐page wrap‐around, but the sense and sign of the change can be deduced by looking at observatories at lower latitude (Hartland or Abinger) as they will typically sense the same direction of change, at the same time, without becoming saturated. A similar test can be made for other types of ambiguities such as inverted signs or to check what missing data may have looked like. With regards to missing data, we suggest the best strategy is to acknowledge and explicitly mark gaps in the record rather than interpolate or infer there is information available. It is helpful to insert flag values to indicate missing values whilst maintaining a consistent time cadence. To enhance the utility of the storm time data we suggest digitizing a period before and after the main phases of the storm to establish what the quiet‐time field level is and to allow alignment with other observatory records. This also ensures that any baseline offsets that are accidentally introduced can be readily detected. The metadata in each the observatory yearbooks also proves an invaluable resource for benchmarking digitized time series with hourly values and daily means.

Manually digitizing a storm is labour‐intensive and requires careful pre‐ and post‐processing of the results. To extract digital traces for a large number of storms would be a very long‐term project. The capability to automate the extraction of the traces using tools such as machine learning would be a great advantage; however, given that much of the metadata lies in machine‐inaccessible formats it is often a case of detective‐like work to be certain that the correct scaling and baseline offsets have been used. Thus an experienced eye is still needed to perform quality‐assurance and validation before release to a public database for scientific use.

## 5 CONCLUSIONS

Over 175 years of continuous magnetic field variation records exist for the UK, starting from Greenwich in the 1840s. Only around 40 years of this data exists in digital format. The remainder of the records consist of images of magnetograms from eight different observatories. Extracting digital values from these images is difficult and requires time and experience to manually trace over the magnetic field changes and convert to time and SI units of nanotesla. Of most interest are large geomagnetic storms in the past, which were they to occur today might potentially pose a hazard to modern grounded infrastructure.

We provide a protocol and methodology for extracting digital values and provide a comparison of the results with a large storm in the digital era (October 2003). We further extract magnetic field variations from storms in 1946 and 1972 to illustrate the difficulties with analogue records but also the benefits of having digital data. We recommend that care be taken in understanding how the old instrumentation and recording systems worked and to avoid common mistakes related to ‘wrap‐around’ of the magnetic traces and from inaccurate application of metadata related to the baseline and variation scaling factors.

## CONFLICTS OF INTEREST

We declare no conflicts of interest.

## AUTHOR CONTRIBUTION

**Ciaran Beggan:** Formal analysis (equal); Investigation (equal); Supervision (equal); Writing – original draft (lead). **Eliot Eaton:** Data curation (equal); Methodology (equal); Visualization (equal); Writing – original draft (equal). **Eleanor Maume:** Data curation (equal); Methodology (equal); Writing – review & editing (supporting). **Ellen Clarke:** Conceptualization (lead); Funding acquisition (lead); Investigation (lead); Supervision (lead); Validation (equal); Visualization (equal); Writing – review & editing (equal). **John Williamson:** Data curation (equal); Methodology (equal); Writing – review & editing (supporting). **Thomas Humphries:** Data curation (equal); Formal analysis (equal); Investigation (equal); Software (supporting); Validation (supporting); Writing – original draft (supporting).

### OPEN PRACTICES

This article has earned an Open Data badge for making publicly available the digitally shareable data necessary to reproduce the reported results. The data is available at https://doi.org/10.5285/2d39fb6c‐debb‐408d‐8abf‐0298eebbf06d. Learn more about the Open Practices badges from the Center for Open Science: https://osf.io/tvyxz/wiki.

## Footnotes

^1^

https://www.bgs.ac.uk/information‐hub/scanned‐records/magnetograms/, accessed 17‐Sep‐2021.

^2^

http://geomag.bgs.ac.uk/data_service/data/yearbooks/yearbooks.html, accessed 17‐Sep‐2021.

^3^

https://largeimages.bgs.ac.uk/iip/magnetograms.html?id=640000/646150

^4^

https://largeimages.bgs.ac.uk/iip/magnetograms.html?id=640000/646452
